# The Combined Use of Automated Milking System and Sensor Data to Improve Detection of Mild Lameness in Dairy Cattle

**DOI:** 10.3390/ani13071180

**Published:** 2023-03-28

**Authors:** Lena Lemmens, Katharina Schodl, Birgit Fuerst-Waltl, Hermann Schwarzenbacher, Christa Egger-Danner, Kristina Linke, Marlene Suntinger, Mary Phelan, Martin Mayerhofer, Franz Steininger, Franz Papst, Lorenz Maurer, Johann Kofler

**Affiliations:** 1 Department of Farm Animals and Veterinary Public Health, University Clinic for Ruminants, University of Veterinary Medicine Vienna, 1210 Vienna, Austria; 2 Department of Sustainable Agricultural Systems, Institute of Livestock Sciences, University of Natural Resources and Life Sciences Vienna, 1180 Vienna, Austria; 3 ZuchtData EDV-Dienstleistungen GmbH, 1200 Vienna, Austria; 4 MSD Animal Health, D18X5K7 Dublin, Ireland; 5 Institute of Technical Informatics, Graz University of Technology, 8010 Graz, Austria; 6 Austria and Complexity Science Hub Vienna, 1080 Vienna, Austria

**Keywords:** lameness, dairy cattle, automated monitoring sensors, automated milking system, locomotion score, claw-position score, early detection of lameness

## Abstract

**Simple Summary:**

The objective of the present study was to develop a tool to detect mildly lame cows by combining already existing data from sensors, automated milking systems (AMSs), routinely recorded animal and farm data and other phenotypes. Ten dairy farms were visited every 30–42 days from January 2020 to May 2021, and locomotion scores (LCSs) and body condition scores (BCSs) were assessed at each visit. For each farm, a lameness incidence risk was calculated. Further, the impact of lameness on the derived sensor parameters was inspected. Finally, random forest models for lameness detection were fit by including different combinations of influencing variables and compared for best results. The best performing model achieved an accuracy of 0.75 with a sensitivity of 0.72 and specificity of 0.78 for predicting an LCS ≥ 2. We conclude that the combination of data from an available sensor device with routinely available AMS data, animal and farm information, and performance records can provide promising results for the detection of mild lameness in dairy cattle.

**Abstract:**

This study aimed to develop a tool to detect mildly lame cows by combining already existing data from sensors, AMSs, and routinely recorded animal and farm data. For this purpose, ten dairy farms were visited every 30–42 days from January 2020 to May 2021. Locomotion scores (LCS, from one for nonlame to five for severely lame) and body condition scores (BCS) were assessed at each visit, resulting in a total of 594 recorded animals. A questionnaire about farm management and husbandry was completed for the inclusion of potential risk factors. A lameness incidence risk (LCS ≥ 2) was calculated and varied widely between farms with a range from 27.07 to 65.52%. Moreover, the impact of lameness on the derived sensor parameters was inspected and showed no significant impact of lameness on total rumination time. Behavioral patterns for eating, low activity, and medium activity differed significantly in lame cows compared to nonlame cows. Finally, random forest models for lameness detection were fit by including different combinations of influencing variables. The results of these models were compared according to accuracy, sensitivity, and specificity. The best performing model achieved an accuracy of 0.75 with a sensitivity of 0.72 and specificity of 0.78. These approaches with routinely available data and sensor data can deliver promising results for early lameness detection in dairy cattle. While experimental automated lameness detection systems have achieved improved predictive results, the benefit of this presented approach is that it uses results from existing, routinely recorded, and therefore widely available data.

## 1. Introduction

Digitalization and automatization have advanced rapidly in the agricultural and livestock sector over the past decades [1,2,3] benefiting both farmers and animals. Further, the use of automated milking systems (AMSs) has increased globally within the dairy industry, automatically and continuously providing farmers with various data outputs concerning milk yield, milk quality, and animal activity [4,5,6,7]. Moreover, sensor technologies are increasingly applied on dairy farms, such as the use of accelerometers for detecting cows in heat [8]. However, progress in breeding and milk performance resulted in high yielding dairy cows with the need for solid herd management to ensure health maintenance. This rapidly highlighted the potential of these technical devices regarding the health monitoring of dairy herds. Nowadays, the use of these sensor data outputs is routinely applied in precision livestock farming. Various sensors are available, and they are either attached to the collar, the ear, leg-mounted as pedometers, or even carried in intraruminal boluses, measuring the rumen’s pH, temperature, and activity [9,10,11,12]. With the aid of these devices, sensor manufacturers have made clear advances in the detection of metabolic and infectious diseases in dairy cows [13].

Alternatively, products to detect lameness routinely and automatically in dairy cattle are still not available on the market. In recent years, many investigators have focused on the development of such systems, but most prototypes are only applied on an experimental setup [14,15,16,17]. Nevertheless, in Austria, lameness is the third most common cause for premature culling of dairy cows with a prevalence of 7.4%, after reproductive problems and mastitis [18]. Furthermore, farmers are expecting an automated lameness detection system associated with low costs and high detection performances. These preconditions must be met before such a tool becomes applicable in practice [17,19,20].

Lameness prevalence in Austria varies greatly between farms, ranging from <5% to approximately 78% with a mean of 36% at the herd level [21,22,23]. However, in most cases, only animals with a LCS of ≥3 (severely lame) are counted as lame, resulting in an even higher lameness prevalence when including mildly lame (LCS = 2) cows [21,22,23]. Lameness in dairy cattle is mainly caused by claw and digital disorders [24], and therefore, lameness has a high impact on animal welfare [25,26]. Painful claw and digital disorders affect the cow’s ability to move around and eat, resulting in a drop in performance. Thus, lameness is often associated with a reduced milk production due to a drop in feed consumption, as well as an impaired fertility, and often leads to premature culling [27,28].

Lameness is commonly detected via visual observation by the farmer and is therefore widely depending on the farmer’s available time and detection skills alongside structural preconditions in the existing barn and work routine [29,30]. However, a visual observation of cows for lameness detection is time-consuming and prone to subjective bias, which impairs the reliability of these data [31,32]. In particular, mild lameness cases at an early stage are easily overlooked by untrained personnel [29,33]. Trying to overcome this issue, systems for automated lameness detection have been developed. Noncontact locomotion scoring systems, working with either force/pressure-sensitive platforms [17,34], or 2D/3D cameras [35,36], deliver objective results without animal interference [37,38]. However, varying detection rates are noted due to walking speed and leg position on platforms [17,39]. A combination with thermal infrared cameras improved detection rates during early lameness stages by capturing the higher temperature from the increasing blood flow [40]. Nevertheless, data processing in this context is complex and time consuming. While these attempts deliver very good results, routine use is still hesitant as farmers are reluctant to invest in such additional and expensive devices [17,41,42].

In contrast, other research approaches focus on automated lameness detection using devices already available on dairy farms. For example, sensors which are directly attached to the cow are frequently used for health monitoring nowadays, by recording behavioral patterns such as rumination, eating, or lying down, relying on the change of these movements for disease detection [2,16,43,44]. Various studies revealed a significant effect of lameness on the cows’ eating time, i.e., the time to secure sufficient dry matter intake, daily milk yield, the number of daily visits to the AMS, and the number of cows that had to be fetched to the AMS, resulting in a higher workload [4,5,45]. A study from Miguel-Pacheco et al. [46] carried out in farms equipped with an AMS showed a significant negative association between total feeding time and lameness as well as the frequency of feeding bouts and lameness. Furthermore, these researchers observed a significant difference in AMS visits between lame and sound cows, with lame animals being 0.33 times less likely to visit the AMS between 00:00 and 06:00. In summary, results from these studies showed that lameness was significantly affecting feeding behavior and AMS visits. All these impacts are likely to have negative consequences for farm profitability, but also for animal health and welfare [25,26,46,47].

The present study was part of the D4Dairy project (https://d4dairy.com/ (accessed on 1 June 2022)), which stands for digitalization, data integration, detection, and decision support in the dairy industry. The primary goal of this project was to integrate data from technology systems already present on dairy farms to build a basis for digital advances in dairy herd management and to enhance animal welfare and sustainable strategies regarding the use of antibiotics, thus ensuring food safety. The aim of the present D4Dairy pilot study was to combine data from animal sensors, AMSs, milk performance testing and farm risk factors to develop a decision support tool for the detection of early stage lameness in dairy cows.

## 2. Materials and Methods

This study was conducted according to the guidelines and institutional ethics of the COMET-Project D4Dairy (project number: 872039) and was discussed and approved by the institutional ethics and animal welfare committee of the University of Veterinary Medicine Vienna on 11 July 2019 in accordance with GSP guidelines and national legislation (ETK-125/07/2019).

### 2.1. Farm Selection

Ten dairy farms located in the Austrian provinces of Lower Austria, Upper Austria, and Styria participated in this trial with a total of 594 cows. Farms were chosen by the following criteria: free stall barns with an AMS and commercially available sensors (SenseHub™, MSD Animal Health, Rahway, NJ, USA). Cows were either already equipped with SenseHub™ dairy sensors or devices were provided by the manufacturer for the study period if the farms were not yet equipped with these sensors. Both the ear tag and collar tag version of sensors were used. Furthermore, participation in the national milk performance recording (Landeskontrollverband—LKV; https://lkv.at/ (accessed on 1 June 2022)) was mandatory.

### 2.2. Animal Data

The LKV provided additional data about animals of these ten farms. Records included animal and farm identification in an anonymized form, age, lactation number, breed, heat events, insemination dates, calving dates, calving difficulty, dry-off date, pedigree, and milk testing records containing milk yield, percentage of fat and protein as well as urea in milk. With the provided fat and protein contents, the fat–protein ratio was calculated. Days in milk (DIM) and the age at first calving were calculated and animals were regarded as purebred cows if the proportion of a single breed exceeded 75%. Breeds comprised four categories including: Fleckvieh (dual-purpose Simmental) (*n* = 368), Brown Swiss (*n* = 50), Holstein Friesian and Red Friesian combined (*n* = 86), and mixed breed (*n* = 90; crossbreed ≥ 25%).

### 2.3. On Farm Data Collection

Employees of the LKV visited each farm every 30 to 42 days from January 2020 to May 2021 during routine milk performance testing. They recorded the locomotion score (LCS) as described by Sprecher et al. [31] and the body condition score (BCS) according to Edmonson et al. [48] (details in Section 2.3.2). These scores were recorded from all cows in milk, dry cows, and heifers during late pregnancy. In addition, all ten farms were visited twice by the first author between November 2020 to March 2021 with 14 days between the first and second visit. During these visits, the LCS and BCS were recorded, and the claw-position score (CPS) was assessed [49,50]. Furthermore, information about farm husbandry, management, and risk factors was documented.

#### 2.3.1. Farm Management and Husbandry

During the first visit, farmers were asked to answer a questionnaire (shown in the Table S1) to assess risk factors including questions on the following topics: general farm information (e.g., conventional or organic) and information on feeding such as feed analyses and calculations of rations. In this context, the level of feed structure and the management of changes in the ration were assessed, i.e., how many days does the farmer allow to ensure the rumen adjustment to new feed rations when changing silage. Furthermore, information about the vitamin and mineral supply of youngstock, dry cows, and lactating cows was recorded. The access to paved outdoor areas was also documented, as well as access to grazing and alpine pasture. Furthermore, the availability of ventilators and other cooling systems in barns was recorded. The integration management of new cows was also a matter of interest, including the presence of calving pens and areas for sick cows. Since all farms were equipped with AMSs, the AMS type was recorded as well as the quality of teat cleansing during the visit. Preventive measurements regarding ketosis, milk fever, and lameness (e.g., routine hoof trimming, hoof trimming intervals, application of foot baths) were noted as well as the number of additional livestock purchases from other farms. Finally, the dimensions of the farm facilities including barn alleys and cubicles were measured and the bedding types, decubital lesions on cows, damages, and slip-resistance of the floors as well as steps or obstacles within the barn were evaluated.

#### 2.3.2. Visual Animal Scorings

In this study, the LCS according to Sprecher et al. [31] was used. Cows were scored from January 2020 to May 2021 during routine milk performance testing while standing and walking to assess back posture and then assigned to a score from 1 to 5 (nonlame to severely lame).

Furthermore, the BCS was calculated using the method described by Edmonson et al. [48] with a scale from 1 to 5, using 0.25-unit increments and therefore resulting in a 17-point scoring system. The BCS was assessed for each cow individually at every visit by a visual examination from the back. A score of 1 indicated an emaciated condition, and a score of 5 referred to an obese animal. The same chart was used for dual-purpose and dairy breeds.

The claw-position score (CPS) is a parameter describing the angle formed by the imaginary interdigital line of each claw pair of the hind limbs and the median line of the cow’s body (the line along the vertebral column) [49,50]. Scoring is done by a visual assessment from the back while the cows stand still, preferably in the feed fence. The angle is caused by differing heel heights between medial and lateral claws on each limb—the higher the heel height of the lateral claw is, the higher the CPS, indicating a greater imbalance of weight distribution between the claws and resulting in a higher risk for the development of claw horn disruption lesions and subsequent clinical lameness. Depending on the angle between the interdigital line and the midline of the body, three scores are differentiated. Score 1 (0–16°) implies a physiological weight distribution between claws; a score of 2 (17–22°) indicates hoof trimming is advised; and a score of 3 (>23°) can be seen as an indicator for subclinical lameness, where hoof trimming is urgently needed before clinical lameness develops [49,50].

#### 2.3.3. Interobserver Reliability

All LKV employees were part of previous studies and were well trained in assessing the LCS and BCS [22,23,51]. To ensure the interobserver reliability for this study, all of them participated in a training on a commercial dairy farm in October 2019 and within two weeks thereafter completed an online video test where different video scenarios of nonlame (LCS 1) and lame cows (LCS 2–5) were presented. For assessing the interobserver reliability, weighted Cohen’s Kappa values were calculated using two different approaches for LCS scoring. First, the 5-level scoring system described above was applied in three testing rounds resulting in a mean weighted Cohen’s Kappa value of 0.65, with a minimum value of 0.55. Reducing the lameness score to a 3-level scale (LCS 1, LCS 2 + 3, LCS 4 + 5), improved Kappa values to a mean of 0.69 (min: 0.55). Combining LCS 1 + 2, LCS 3, and LCS 4 + 5 gave the best results, with a mean weighted Kappa value of 0.72 (min: 0.61). However, the authors refrained from merging nonlame cows with mildly lame cows as this study aimed at the detection of lameness during its early stages with the aid of behavioral differences registered by sensors. Therefore, LCS 1 was further referred to as LCS-G 1, LCS 2 and 3 were combined to LCS-G 2, and LCS-G 3 comprised LCS 4 and LCS 5 scorings. Weighted Cohen’s Kappa values >0.6 are considered to indicate a high agreement, values between 0.6 and 0.4 indicate a moderate agreement, and values below 0.4 indicate a low interobserver agreement [52].

For the BCS, two test rounds were performed and overall, a good agreement between all participating LKV employees was determined, resulting in a mean weighted Kappa of 0.81 (min: 0.61) in round 1 and 0.78 (min: 0.61) in round 2.

### 2.4. AMS and Sensor Data Collection and Transfer

The data from the AMSs (Lely Astronaut A4 and Lely Astronaut A5, Lely International N.V., Maassluis, The Netherlands; DeLaval VMS, DeLaval, Tumba, Sweden; GEA Monobox, GEA Farm Technologies, Bönen, Germany) were provided by the manufacturers. After validation for missing records and implausible milk quantities, the final AMS dataset included the total daily milk yield (DMY), number of milkings per day, milking interval, and the average milk production per hour. AMS parameters that were regarded as significant for lameness occasions in other studies were not included in the transferred dataset by the AMS data providers. Examples included refusals (visits at the AMS without milking), concentrate left-overs, and milking duration.

Sensor data from the SenseHub™ dairy system were available for eight farms during the whole observation period from July 2020 to May 2021. The other two farms only provided 6–8 months of sensor data due to a delay in the installation during COVID-19. The SenseHub™ dairy sensors are automated monitoring sensors administered via ear or collar tags which record cow activities and transmit them via an installed antenna every 20 min. Sensor data were then preprocessed by the manufacturer and provided by MSD Animal Health. The dataset included the parameters ‘Rumination’, ‘Eating’, ‘Walking’, ‘Rest’, ‘Activity_Mid’, ‘Activity_High’, ‘Over_Heat’ and ‘Reserved’ and were stated for every hour as minutes per hour. The parameter ‘Rest’ registers low activity, such as lying down. ‘Activity_Mid’ comprises behavior with medium activity levels, for example drinking, using the cow brush, walking around, interacting with other cows. ‘Activity_High’ identifies running around or intensive movements during heat. ‘Walking’ was rarely detected in our dataset, with only 0 to 2 min per hour. ‘Over_Heat’ records heat stress via heavy breathing. ‘Reserved’ is associated with animal behavior that has not been defined up to this study. Further, the calculated index ‘Activity_Trend’, which combines the duration and intensity of activity, was transmitted.

### 2.5. Annual Milk Yield and Lactation Numbers

Overall, ten dairy farms participated in this study with a total of 594 cows. The annual herd milk performance in 2020 was 9001 kg with a range from 6367 kg to 10,496 kg. The number of dairy cows on each farm ranged from 46 to 84 with a mean of 59.40 cows per farm. In total, 61.95% of animals were Fleckvieh, 14.48% Holstein Friesian, 8.42% Brown Swiss, and 15.15% of participating cows were of mixed breed.

Lactation numbers varied between first and tenth lactation, with 29.34% of records after first calving, 26.55% after the second, 15.54% in third lactation, 11.18% during fourth lactation, and 17.39% during fifth and higher lactation. The lactation number was highly significant (*p* < 0.0001) when comparing LCS groups, as well as the age of the animals (*p* < 0.0001). Furthermore, the number of days in milk was not significant for lameness events (*p* = 0.635). The age of the dairy cows at first calving ranged from 19 to 43 months with a mean of 29.13 months. Within LCS groups, the LS means for the age at first calving were 29.0 (±0.09), 29.3 (±0.11), and 29.3 (±0.21) months for LCS-G 1, 2, and 3, respectively. However, the age at first calving was not significant for lameness (*p* = 0.132).

### 2.6. Farm Management and Husbandry Conditions

According to the farmer questionnaires, all animals were kept in free-stall barns and milked by AMSs (five De Laval VMS, two Lely Astronaut 4, one Lely Astronaut 5 and two GEA Monobox). Nine farms were conventional, one farm was organic. Two farms were equipped with slatted floors, one with partly slatted floors and partly solid concrete floor, four farms with solid concrete floors, and three with solid concrete floors covered with rubber mats. Eight facilities used straw for cubicle bedding, two farms used separated manure with one of them also offering a lying area with compost. Only one farm offered pasture for their milking cows and four facilities for their dry cows. Cows had permanent access to outdoor areas in four herds. Youngstock was kept on seasonal pasture on five farms, three of them also offering alpine grazing. Six barns were equipped with cooling systems for dairy cattle, such as ventilators or showers. Hoof trimming by professional hoof trimmers was performed twice a year on all farms on all cows in milk and on one farm also on lame youngstock. If acute lameness cases occurred between these visits, they were treated by the farmer and/or hoof trimmer.

On four operations, damaged flooring was noted, as well as slippery floors in two farms. In eight farms, obstacles and steps within the barn, especially around the AMS, were observed. Feeding alleys had a mean width of 436.50 cm ranging from 320 to 600 cm. Additional walking alleys were measured at mean of 325 cm (250–450 cm). On each farm, the dimensions of four different cubicles were measured including the cubicle bed length (mean: 189.15 cm), cubicle width (mean: 125.00 cm), height of the neck rail (mean: 115.00 cm), neck rail diagonal (mean: 193.60 cm), brisket board (mean: 22.67 cm), curb (mean: 23.85 cm), and bedding height (mean: 8.79 cm).

### 2.7. Statistical Analyses

Prior to the analysis, all data were cleaned, preprocessed, and further anonymized by ZuchtData EDV-Dienstleistungen GmbH (Vienna, Austria) to guarantee data privacy, as agreed on by all project partners. Further data processing, descriptive statistics and statistical modelling were executed in R Statistical Software (v4.1.2, R Core Team, Vienna, Austria) [53].

#### 2.7.1. Lameness Incidence Risk

Locomotion scoring was done every 30 to 42 days during milk performance testing. Therefore, every record of lameness during these scorings was qualified as a newly diseased animal. The lameness incidence risk was calculated by dividing the sum of all new cases of lameness during the study period by the total number of scored cows during the whole study period. The total lameness incidence risk was calculated by including all animals, regardless of their equipment with additional sensors or without. In addition, the correlation between the LCS and CPS was calculated using the Spearman’s correlation coefficient *rs* for ordinal data.

#### 2.7.2. Impact of LCS on Sensor and AMS Parameters

According to the results of the interobserver reliability assessment, the five levels of locomotion scores were combined to the three levels as described in Section 2.3.3.

The sensor parameters ‘Reserved’, ‘Walking’, ‘Over_Heat’, and ‘Activity_High’ were removed from further analysis due to irrelevance or low detection rate. The remaining sensor parameters were ‘Rumination’, ‘Eating’, ‘Activity_Mid’, ‘Rest’, and ‘Activity_Trend’. Hourly activity parameters were summed up for each day, and for the index ‘Activity_Trend’, the daily mean was calculated. Subsequently, sensor records from the day of locomotion scoring were used for further analysis.

The sensor and AMS parameters were analyzed by linear models (R package lsmeans (v2.30-0) [54]), including the following effects: farm, breed, lactation number, lactation stage, age at first calving, and LCS-G. Furthermore, the least-squares (LS) means and standard errors (SE) for each LCS-G were calculated. A Tukey–Kramer post hoc test was then performed to calculate the *p*-values for each LCS-G comparison.

Furthermore, the animal behavior was graphically presented as a function of daytime to inspect the hourly impacts of lameness on the recorded sensor parameters (shown in Figure S1). Lame cows tend to avoid conflicts over resources and therefore behave contrarily to sound cows [55]. A previous study by Miguel-Pacheco et al. [46] showed significant differences in AMS visits between healthy and lame cows between 00:00 and 06:00. In line with these results, time between 08:00 to 20:00 was considered daytime and sensor parameters were explicitly summed for these hours. Parameters during nighttime were not considered separately, as all animals behaved similarly during the night.

#### 2.7.3. Lameness Detection with Random Forest

Lameness detection was carried out with a machine learning approach using a random forest approach [56,57,58,59]. This algorithm is implemented in the R packages ranger (v 0.14.1) [60] and caret (v6.0-88) [61], which were used in this study. The idea of this procedure is to improve the performance of a single decision tree by averaging multiple individual decision trees to minimize their own errors [56,57,58]. To further improve the performance of our models, a 10-fold cross-validated hyperparameter tuning was conducted. By doing so, the best number of variables tried at each split was determined, with constant values for the number of trees (*n* = 500) and minimum node size (*n* = 1).

Results for the different model approaches were evaluated by statistical performance metrics including accuracy, sensitivity, and specificity. The mean and standard deviation of these performance metrics were stated for all cross-validation procedures. Model performance was then compared using the accuracy. Key figures were calculated as followed with numbers of true positives (TP), true negatives (TN), false positives (FP), and false negatives (FN):

$$Sensitivity=\frac{TP}{TP+FN}$$

$$Specificity=\frac{TN}{TN+FP}$$

$$Accuracy=\frac{TP+TN}{TP+TN+FP+FN}$$

##### Model Parameters

The desired outcome for our final model was a prediction of whether a cow was not lame (LCS-G 1) or lame (LCS-G 2 + 3). Not all cows in the dataset were equipped with additional sensors, thus the number of cows dropped from 593 to 374 with 2682 observations. Overall, 52.27% of these records belonged to nonlame cows and 47.73% to lame cows, resulting in a balanced dataset for disease detection. Therefore, no over- or undersampling procedures were implemented. Scorings of dry cows and heifers were removed from the dataset to only include records with AMS data. A detailed description of the parameters used for the detection models is given in Table 1. The z-score for continuous sensor parameters was calculated. The z-score is a standard score measuring by how many standard deviations the initial record in our dataset differs from the overall mean of the herd. Furthermore, the parameters were also scaled to investigate how much the value on scoring days differed from the mean of each individual animal during the study period. Both scores were calculated for the daily sum of each parameter, as well as for the sum of activity records during daytime. For the model calculation, the difference in BCS from one scoring day to the previous scoring day was used.

##### Lameness Detection Model with Animal-Based Split

In the first approach, an animal-based split [62] was chosen, meaning that all observations on an individual cow were either placed within the training dataset or test dataset. The dataset was randomly split by animal into a training dataset containing 80% of the records and a test dataset consisting of the remaining 20%. The model was then trained on the larger training records, and disease detections were made for the test dataset. Five different models were built with varying variable combinations, as shown in Table 2. A fourfold cross validation was conducted for each model. For the training of model 5, only days with LCS and CPS scorings performed by the first author were considered for cows in milk, yielding 574 records.

##### Lameness Detection Model with Farm-Based Split

In the second approach, a farm-based split [63] was used. The dataset included the same data and variables as in Model 3 of the animal-based split approach (Table 2) and was split into ten sub-datasets according to the ten farms, as the idea was to develop a tool that could detect lame cows for one farm based on training information from other farms, even without additional recorded phenotypes such as the BCS and CPS. For each round, nine farms were in one training data set, and predictions were made for one farm as the test dataset, resulting in a 10-fold cross-validation. In the next step, the dataset was split into three groups according to lameness incidence risk groups (<30% (4 farms), 30–50% (3 farms), >50% (3 farms)). In each group, one farm was defined as the test data and the remaining farms were used for model training. This procedure was repeated for every farm, resulting in a 3- and 4-fold cross-validation, respectively. Performance metrics were calculated for overall results as well as within certain lameness incidence risk groups (<30% (4 farms), 30–50% (3 farms), >50% (3 farms).

## 3. Results

The number of farms participating in this study, the number of cows per farm, the mean annual milk yield and the distribution of cows in terms of their lactation numbers were mentioned earlier.

### 3.1. Lameness Incidence Risk

A total of 8285 locomotion scores were recorded over eleven months from July 2020 to May 2021, including all dairy cows on the farms, either in milk or dry, as well as heifers during late pregnancy. Combining all farms, 55.93% of records were LSC 1 (nonlame cows: *n* = 4634), 3052 records (36.84%) belonged to LCS-G 2, and 599 records (7.23%) to LCS-G 3. LCS groups by breed illustrated a numerically high occurrence of lame cows for the breed Fleckvieh, which is the main breed in Austria and therefore also had the highest number of lameness scorings in the dataset (Table 3). However, only 49.62% of the 5298 records were LCS-G 1 during the whole observation period. In comparison, 71.53% of scorings for Brown Swiss cows were LCS-G 1, 69.98% of Holstein cows and 61.97% for mixed breed cows. For LCS-G 2, Fleckvieh cows also showed the highest incidence with 41.15%, followed by mixed breed (34.29%), Holstein (27.19%) and Brown Swiss (23.56%) cows. A high rate of Fleckvieh cows (9.23%) were distinctly to severely lame (LCS-G 3). Results for other breeds regarding LCS-G 3 were comparatively low, ranging from 2.83% for Holstein to 4.91% for Brown Swiss cows.

Lactation numbers were divided into one, two, three, four, and five or more lactations (Table 4). A continuous increase of lame cows with rising lactation numbers was observed. During the first lactation, 70.97% of the observations were LCS-G 1, during the second lactation 60.54%, the third 48.58%, the fourth 45.39%, and during the fifth and higher lactations, 34.11%. Similarly, a steady increase was determined for LCS-G 2, from 25.73% after the first calving to 52.29% after the fifth or higher calving. LCS-G 3 records increased from 3.30% to 13.60%. Apparently, the total number of observations was less for the lactation number due to the exclusion of scorings before the first calving. The total number of cows was considerably higher, as most cows reached a higher parity during the study period and therefore appeared in two categories.

In general, however, lameness incidences differed very much between farms (Table 5). On four farms (A, B, F, H) the lameness incidence risk was quite similar with a high number of nonlame cows (71.12–72.93%). The highest numbers for severely lame animals (LCS-G 3) were recorded on farms C, E, and J with a range of 12.22–13.95%. The lowest value was reached on farm I with 0.84%.

### 3.2. Body Condition Scoring

**Figure 1:**
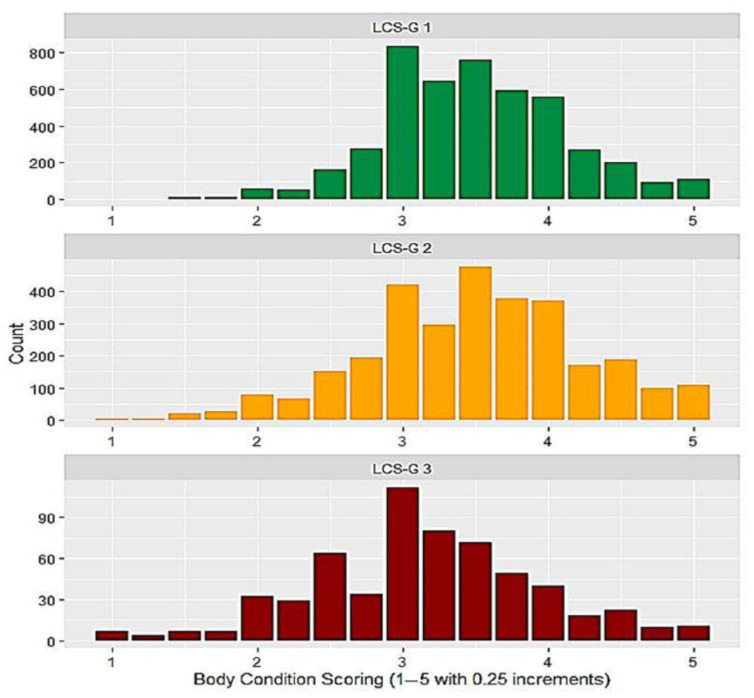
Distribution of body condition scores according to LCS-G 1–3; LCS-G 1–3: locomotion score groups 1 to 3.

A total of 8275 body condition scorings were recorded, using the same chart for all breeds. The BCS distribution according to each LCS-G is presented in Figure 1.

### 3.3. Claw-Position Scoring

During the first author’s farm visits, the CPS was assessed for 593 cows, resulting in a total of 1186 observations. Table 6 shows the distribution of the CPS according to the LCS-G of each observed cow. A steady decrease of CPS 1 was also reflected in higher locomotion scores, thus, numbers for CPS 2 and 3 increased with LCS. Furthermore, Spearman’s correlation coefficient rs for ordinal data was calculated and indicated a weak positive correlation between LCS-G and CPS (*rs*: 0.394) with a high significance (*p* < 0.0001).

### 3.4. LCS-G and AMS Data

Data from the AMS systems provided information about the animal ID, the day and time of milking, and the amount of milk yield. The dataset was validated for missing milking records prior to further preprocessing. The additional parameters average milk production per hour and daily milk yield were calculated. The detailed results are shown in Table 7.

The LS mean for the number of daily milkings for LCS-G 1 cows was 3.24 (±0.03), 3.14 (±0.03) for LCS-G 2 cows, and 2.93 (±0.06) for LCS-G 3 cows. The differences in daily milking events differed significantly between LCS-G 1 and LCS-G 2 (*p* = 0.0291) as well as LCS-G 2 and 3 (*p* = 0.0018) and were highly significant between LCS-G 1 and 3.

The mean daily milk yield was 26.7 kg (±0.27 kg) for nonlame cows, 26.5 kg (±0.31 kg) for LCS-G 2 cows, and 25.7 kg (±0.57 kg) for LCS-G 3 cows. This resulted in no significant differences for DMY (*p* = 0.1913). Significant results (*p* = 0.0128 and *p* = 0.0001) were obtained for the comparison between LCS-G 1 and 2 as well as LCS-G 2 and 3 for the parameter milking interval. *p* values for nonlame cows and severely lame cows were highly significant. No significant differences between LCS groups were observed for hourly milk production (*p* = 0.3300).

### 3.5. LCS-G and Sensor Parameters

The results from the linear models for the sensor variables are shown in Table 8. Overall, no significant differences for ‘Rumination’ were observed between LCS groups, with an LS mean for rumination time for nonlame cows of 560 min per day, 561 min for mildly lame cows, and 561 min for severely lame cows. The sensor parameter ‘Eating’ delivered significant results for total daily eating time for all comparisons. Sound, nonlame cows spent on average 281 min per day eating, mildly lame cows 268 min, and severely lame cows 245 min. Hence, lameness resulted in a drop in eating time by approximately 13 or 36 min, respectively.

Records for the sensor parameter ‘Rest’ showed highly significant differences between LCS groups: the mean for LCS-G 1 was 387 min per day, 410 min for LCS-G 2, and 442 min for LCS-G 3 animals. Thus, time spent with low activities increased by 23 and 55 min for LCS-G 2 and 3, respectively, when compared to nonlame cows.

‘Activity_Mid’ is a sensor parameter consisting of activities with medium intensity, such as walking, interacting with other cows, and using the cow brush. Nonlame cows spent on average 148 min per day with medium intensity activities, LCS-G 2 cows 140 and LCS-G 3 cows 128. *p*-values were significant for all LCS-group comparisons.

‘Activity_Trend’, an index for the duration and intensity of activity, was provided as the mean value for the scoring day. Results were significant (*p* = 0.0002) to highly significant for group comparisons.

### 3.6. Detection of Lameness Using a Random Forest Algorithm

#### 3.6.1. Lameness Detection Model with Animal-Based Split

In the first model of this approach, only sensor parameters were used to detect lame and nonlame animals. Results were comparatively low for all performance metrics with a mean accuracy of 0.623 (±0.006), a mean sensitivity of 0.610 (±0.009), and a mean specificity of 0.640 (±0.005).

In the second model, animal and farm data were combined with AMS data and performance records with similar results for the accuracy (0.629 (±0.020)), a higher sensitivity (0.657 (±0.022)), and a lower specificity (0.605 (±0.020)). However, better results were achieved for the third model, combining farm and AMS data with sensor variables (accuracy: 0.680 (±0.014), sensitivity: 0.695 (±0.030), specificity: 0.668 (±0.041)). Adding BCS to the data in Model 4 resulted in a boost in specificity to 0.701 (±0.031), in sensitivity to 0.738 (±0.014), and an overall accuracy of 0.719 (±0.010). The highest accuracy of 0.753 (±0.046) was achieved with Model 5 (including CPS), resulting in a slightly lower sensitivity of 0.725 (±0.090) and the highest specificity of 0.775 (±0.025) (Table 9).

#### 3.6.2. Lameness Detection Model with Farm-Based Split

Training the random forest algorithm on a farm-based split of the dataset of Model 3 resulted in an overall accuracy of 0.605 (±0.060) with a maximum of 0.712 and a minimum of 0.522. Sensitivities ranged from 0.421 to 0.771 with a mean of 0.613 (±0.131) and specificities ranged from 0.355 to 0.802 with a mean of 0.580 (±0.117).

Looking more closely at individual farms, results suggested that the model worked best on farms with low lameness incidence risks (mean 27.97%), whereas farms with high incidence risks of 49–65.52% had poorer predictive outcomes. Taking the lameness incidence risk into account, performance records for each of the three lameness incidence risk groups are stated in Table 10.

In the next step, model training was performed within these lameness incidence risk groups. Therefore, farms were split into three sub-datasets according to their lameness incidence risk (<30% (four farms), 30–50% (three farms), >50% (three farms)). Results are shown in Table 11. By doing so, the model performance, as evaluated by the accuracy, was improved for two categories (<30% and >50%) compared to the training on all farms.

## 4. Discussion

In contrast to many other studies that chose an experimental approach [14,43,64,65], the aim of the present study was to combine already existing data from different sources (sensors, AMS, animal, and farm information) in dairy farms to identify cows during early lameness stages. Furthermore, a locomotion score of two was considered because it indicates mild lameness [31] and our objective was the detection of early lameness stages. In other studies, however, cows with a mild lameness were often grouped in the ‘nonlame’ group and only cows with LSC ≥ 3 were used for statistical analyses [16,21,64,65,66].

The locomotion score records of all cows on the ten participating farms, including cows in milk and dry cows, as well as heifers during late pregnancy, resulted in an overall mean lameness incidence risk of 44.08% with a range from 27.07 to 65.52%. The lameness incidence risk for Fleckvieh cows was overall high (50.38%) when also counting LCS 2 as lame. In recent reports from Austria [22], including 144 herds, and from North America [67], the mean lameness prevalence including LCS 2 ranged from 33.6% to 63.0%.

With a share of approximately 75%, Fleckvieh is the main breed in Austria [18,47] and was therefore overrepresented in our study. Furthermore, it is the only dual-purpose breed in this study. While Fleckvieh cows can reach milk yields similar to other breeds involved [22], these cows are commonly much heavier than, for example, Holstein cows. Moreover, Fleckvieh cows reached higher lactation numbers in our study and thus contributed to the majority of cows with five or more lactations as compared to Holstein cows [18,68]. Nevertheless, lameness incidence risks were high on most participating farms, highlighting an ongoing issue in some dairy herds. However, this is not representative for all Austrian dairy farms, as data from a recent benchmarking study for claw health suggested that many dairy farms in Austria have a good claw health status and lameness incidence rates of less than 24.6% for the herds in the 25th percentile [23].

Aside from the technical functionality of the provided automated monitoring sensors, the transfer of data and processing is very important to consider [17]. These sensors are originally used for heat detection and the sensing of metabolic or infectious diseases. Thus, the data processing and algorithm training are designed for this purpose. For example, sensors used in this study could not provide information about behavior such as walking or lying down. As shown in other studies, the duration of lying bouts as well as total lying time are significantly higher for lame cows [5,39,55,69]. Further, counting steps by pedometers can also improve disease detection [8,16]. Moreover, the parameter ‘Reserved’ is compiled of various sensor records that could not be linked to a certain animal behavior up to this day. Especially in this context, identifying certain movement patterns could be crucial for the substantial improvement of automated lameness detection in an already implemented sensor system. Similar to findings by Garcia et al. [4], lame animals in the present study showed a differing activity pattern during daytime as compared to nonlame cows, but as opposed to their results, we recorded the lowest activities for lame cattle during the early morning (5:00–6:00 am). As reported in other studies, total rumination time did not differ between LCS groups [69,70], while time spent eating dropped by 13 and 36 min for LCS-G 2 and LCS-G 3, respectively, compared to nonlame animals. This can be explained by an up to 40% faster eating rate for lame cows [70,71].

Transferred AMS records provided the number of daily milkings, milking interval, the amount of milk, and the average milk production per hour to correct for missing values and irregular milking patterns. However, the average milk production per hour ranged from 1.20 to 1.23 and was not significant for lameness events in this study. On average, nonlame cows visited the AMS 3.24 times per day. LCS-G 2 animals recorded a significant (*p* = 0.0291) lower mean of 3.14. The difference for LCS-G 3 was noted as highly significant with mean visits of 2.93 when compared to LCS-G 1. Similar to this, Miguel-Pacheco et al. [46] found a significant reduction from 3.2 daily milkings to 2.8 milking events when comparing nonlame and lame animals. Similarly, the milking interval was also significant to highly significant for group comparisons.

A lameness detection approach using a random forest algorithm [56,57,58,59] was applied in this study. Lameness detection solely relying on sensor parameters resulted in the lowest accuracy of 0.623 (±0.005) with a sensitivity of 0.610 (±0.009) and a specificity of 0.640 (±0.030). A study by Borghart et al. [2] found similar results for lameness detection using parameters from a different sensor system (MooMonitor+^®^, Dairymaster^®^, Causeway, Ireland) with our model outperforming in terms of specificity (64% versus 53%).

Using animal and farm data combined with AMS data and performance measurements resulted in an accuracy of 0.629 (±0.020), a sensitivity of 0.657 (±0.022), and a specificity of 0.605 (±0.020). However, detection results improved by combining all these available data sources (accuracy = 0.680 (±0.014), sensitivity = 0.695 ± 0.030, specificity = 0.668 ± 0.041). In the next step, the difference from body condition scores from one scoring day to the previous scoring day was added. This boosted our model results to an accuracy of 72%, a sensitivity of 74%, and a specificity of 70%. The findings by Borghart et al. [2] showed a better sensitivity (78%) and specificity (78%). However, their final model included the live body weight, while our study relied on an estimation of the cow’s condition using the BCS. Further, in the study of Borghart et al. [2], only Holstein Friesian cows from one single farm were used, while the present approach combined the data of ten farms with dairy cows belonging to different breeds. In our case, the same chart was used to score all cows. Especially on farms with Fleckvieh and Holstein Friesian cows, the dual-purpose breed tends to be overestimated during the scoring of the body condition [72]. The best detection model was obtained when the recorded phenotype CPS was added. The accuracy amounted to 75%, the sensitivity to 72%, and the specificity to 78%. Although the sensitivity was lower compared to that of Model 4, the specificity was distinctly raised. In the present study, AMS data slightly contributed to improving the lameness detection. However, only the time of milking and the amount of milk were provided in the original AMS dataset, with available records containing DMY, number of milkings, time between milkings, and average milk production per hour in the validated dataset. Other studies using AMS data reached better results (sensitivity 79%, specificity 83%) by including animal refusals (times the cow visits the AMS without being entitled to be milked), concentrate left-overs, milking duration, and average milk flow [4,73]. As these parameters were not included in the transferred AMS data, adding these records to the final dataset could potentially boost prediction results. A study by Lasser et al. [74], using animal and farm data to predict lameness in dairy cows, also highlighted the importance of appropriate machine learning selection. They demonstrated that more advanced techniques (e.g., XGBoost [75]) did not generally outperform other approaches. In their case, a logistic regression reached an accuracy of 89%, a random forest model 87%, and XGBoost 81%. Therefore, the underlying dataset and a suitable algorithm should always be evaluated [74].

The highest specificity in the present study was achieved by including the CPS (specificity = 0.775 ± 0.025), albeit with a drop in sensitivity. However, the exclusion of cows with no sensor equipment and dry cows resulted in only 574 remaining observations. Nevertheless, the clinical trait CPS may improve the identification of cows that are at a higher risk of becoming lame in the future due to an uneven weight distribution between lateral and medial claws on the hind limbs. The CPS is applied in Austria by some veterinarians and farmers routinely for monitoring subclinical lameness, but publications on this topic are rare [49,50]. In a recent study evaluating alternatives to locomotion scoring for detecting lameness, one of the applied indicators during the in-parlor scoring procedure was ‘overgrown’ hoofs, indicated by different heel heights of the lateral and medial claws [76].

Using a farm-based split instead of an animal-based split on Model 3 reduced the mean accuracy to 0.605 (±0.060). This could be because an animal-based split can pass some relevant information (e.g., herd management practices) from the training to the test set. Therefore, a certain overfit cannot be excluded. This is in accordance with Lasser et al. [74], where animal and farm-based splits were also compared. However, on some farms, accuracy yielded comparatively high values of 0.72, 0.67, and 0.65. On the other hand, values decreased to 0.53 and 0.52 in other farms. Notably, poorer detection results for farm-based splits occurred primarily on those farms with high lameness incidence risks (farms C, D, E, G, I, J). The lameness incidence risk on these six farms had a mean of 53.15% with a range of 41.13 to 65.52%. In contrast, the remaining four farms with good predictive results had a mean incidence risk of 27.97%, with a minimal range of 27.07 to 28.88%. Results for farm G were among the lowest in all approaches. However, this was the only organic farm in the present study and operated with mostly different husbandry and management measurements including low-input strategies (e.g., in comparison low concentrate intake, grazing, high calving intervals from only sometimes presenting a breeding bull and no artificial insemination, partly cow-bound rearing). Model training within the lameness incidence risk groups slightly improved the results for disease detection in two categories. The accuracy for the lameness incidence risk <30% changed from 0.651 (±0.061) to 0.687 (±0.020), for 30–50% from 0.598 (±0.022) to 0.591 (±0.015), and for >50% from 0.553 (±0.042) to 0.574 (±0.064).

Our results demonstrate that the performance of this approach depends on the similarities of farm conditions in the training data with those of the test data. For improving disease detection under different farm conditions, a further inclusion of various other data (e.g., differing management measurements regarding hoof trimming, data from conformation scoring, varying husbandry conditions, data from organic farms with pasture), from these farms would be necessary in the future. However, the bases for such big data analyses were already implemented within the D4Dairy project (https://d4dairy.com/ (accessed on 1 June 2022)) by harmonizing data standards and creating interfaces for data integration in the future.

Another challenge we faced during this study was the individual animal behavior in connection with lameness as reported also by others [15,69]. On the one hand, individual behavior (‘Rumination’, ‘Eating’, ‘Rest’, ‘Activity_Mid’, ‘Activity_Trend’) was scaled for comparison with other animals; on the other hand, the deviation for each individual cow was also considered. However, some moderately to severely lame cows reacted with a visible change in movement patterns, while other cows seemed completely unphased by painful digital disorders. Conversely, some animals with a prolonged lameness history did not change their behavior visibly after successful treatment, which may be explained by the presence of chronic lameness and induced pain memory [26,77].

Lameness incidence risk increased with the lactation number according to our data and according to the literature [22,66,78]. Nevertheless, dairy cattle with higher lactation numbers and higher milk yield recorded less differences in sensor parameters. This could imply that farmers selected cows with a higher behavioral tolerance towards lameness despite an increasing age, meaning that affected cows are still productive while being or not successfully treated. This suggests that recorded aspects (especially ‘Eating’, ‘Rest’, ‘Activity_Mid’, ‘Activity_Trend’) provided by sensors can add valuable information for lameness detection but may be limited by individual behavioral reactions to painful digital diseases [15,69].

There were also some limitations in this study. As not all cows on participating farms were equipped with additional monitoring devices, sensor data were recorded from 374 cows only, instead of all 594 animals present on the farms. Farmers were allowed to freely choose which animals to equip with these ear or collar tags. This aspect possibly led to a biased selection of particularly valuable and high-yield cows. Moreover, as herd sizes in Austria are comparatively small [18,21], farm selection criteria resulted in quite similar husbandry and management circumstances. This led to an exclusion of many risk factors that were regarded as highly correlated to lameness in other studies [79,80,81]. Other approaches including the assessment of risk factors regarding feeding, housing, husbandry, and management measures have made clear advances in lameness detection and may be beneficial for a further combination of different data sources [81].

## 5. Conclusions

For identifying lame dairy cows during early lameness (mild) stages that may easily be overlooked during daily routine, the records of a commercially available sensor device (attached to the collar or ear) were combined with routinely available data (AMS data, animal and farm information, performance records) and visually assessed animal scorings (LCS, BCS, CPS). The best performing model achieved an accuracy of 75% (sensitivity: 72%, specificity: 78%). Although specialized automated detection systems have achieved higher detection rates in experimental setups, their practical implementation is rare. Instead, a combination of these widely available data records can deliver satisfactory results for lameness detection in dairy cattle. As automated monitoring sensors and AMSs are becoming more frequent, the evaluation of a larger data collection with a wider variation of farms could possibly improve this method for the detection of early stage lameness in the future.

## Figures and Tables

**Table 1:** Parameters that were included in the final detection models.

Parameter	Description
	Animal data
age	In months between date of birth and scoring date
breed	Fleckvieh, Brown Swiss, Holstein Friesian, mixed breed
lac_num	Lactation number 1, 2, 3, 4, 5+
DIM	Days in milk
calving_age	Age at first calving in months between date of birth and first calving
	Routinely recorded performance data
milk_herd	Annual herd milk performance of 2020
milk_protein	Protein content in milk
milk_protein_prev	Protein content in milk of previous performance testing
milk_urea	Urea content in milk
milk_urea_prev	Urea content in milk of previous performance testing
milk_fat_protein_ratio	Fat–protein ratio in milk
milk_fat_protein_ratio_prev	Fat–protein ratio in milk of previous performance testing
BCS_diff	Difference in BCS from one scoring day to the previous scoring day
CPS	Claw-position score from 1 to 3
	AMS data
ams_DMY	Daily milk yield in kg
ams_hourly_production	Milk production per hour
ams_num_milkings	Number of daily milkings
ams_interval_milkings	Time between milkings
	Sensor data
Rumination	Total daily rumination time, scaled by mean of herd at scoring day
Eating	Total daily eating time, scaled by mean of herd at scoring day
Rest	Total daily time with low activity, scaled by mean of herd at scoring day
Activity_Mid	Total daily time with medium activity, scaled by mean of herd at scoring day
Activity_Trend	Calculated daily mean for index Activity_Trend, scaled by mean of herd at scoring day
Rumination_daytime	Minutes spent ruminating during daytime, scaled by mean of herd at scoring day
Eating_daytime	Minutes spent eating during daytime, scaled by mean of herd at scoring day
Rest_daytime	Minutes with low activity during daytime, scaled by mean of herd at scoring day
Activity_Mid_daytime	Minutes with medium activity during daytime, scaled by mean of herd at scoring day
Activity_Trend_daytime	Mean Activity_Trend index during daytime, scaled by mean of herd at scoring day
Rumination_animal_daytime	Minutes spent ruminating during daytime, scaled by mean of individual animal at study period
Eating_animal_daytime	Minutes spent eating during daytime, scaled by mean of individual animal at study period
Rest_animal_daytime	Minutes with low activity during daytime, scaled by mean of individual animal at study period
Activity_Mid_animal_daytime	Minutes with medium activity during daytime, scaled by mean of individual animal at study period
Activity_Trend_animal_daytime	Mean Activity_Trend during daytime, scaled by mean of individual animal at study period
	Risk factors
Results from the farmer’s questionnaire were evaluated and risk factors were added to the analysis. Remaining risk factors that were used in the model are shown in the Supplementary Materials (Table S1, Figure S1).	

**Table 2:** Four different models with varying combinations of variables were built.

Model 1	Model 2	Model 3	Model 4	Model 5
Sensor data	Animal data Milk performance records Farm risk factors AMS data	Animal data Milk performance records Farm risk factors AMS data Sensor data	Animal data Milk performance records Farm risk factors AMS data Sensor data BCS difference	Animal data Milk performance records Farm risk factors AMS data Sensor data CPS

**Table 3:** Lameness incidence risk on all 10 farms according to breed and for all animals; LCS-G 1–3: locomotion score groups 1 to 3.

Breed	Total Cows	Total Observations	LCS-G 1 (%)	LCS-G 2 (%)	LCS-G 3 (%)
Fleckvieh	368	5298	49.62	41.15	9.23
Holstein	86	1096	69.98	27.19	2.83
Brown Swiss	50	713	71.53	23.56	4.91
Mixed breed	90	1178	61.97	34.29	3.74
All breeds	594	8285	55.93	36.84	7.23

**Table 4:** Lameness incidence risk according to lactation number; LCS-G 1–3: locomotion score groups 1 to 3.

Lactation Number	Total Cows	Total Observations	LCS-G 1 (%)	LCS-G 2 (%)	LCS-G 3 (%)
1	298	2394	70.97	25.73	3.30
2	289	2167	60.54	33.55	5.91
3	185	1268	48.58	42.43	8.99
4	122	912	45.39	45.51	9.10
5+	124	1419	34.11	52.29	13.60
	*n* = 1018	*n* = 8160			

**Table 5:** Lameness incidence risks according to the ten farms; LCS-G 1–3: locomotion score groups 1 to 3.

Farm	Total Cows	Total Observations	LCS-G 1 (%)	LCS-G 2 (%)	LCS-G 3 (%)
A	65	790	71.27	23.92	4.81
B	58	665	72.93	24.67	2.40
C	55	913	51.00	35.33	13.67
D	46	525	34.48	60.76	4.76
E	84	1391	43.78	42.27	13.95
F	51	735	72.79	23.81	3.40
G	58	727	42.64	53.65	3.71
H	46	599	71.12	25.21	3.67
I	70	958	58.87	40.29	0.84
J	61	982	50.31	37.47	12.22
	*n* = 594	*n* = 8285			

**Table 6:** Distribution of CPS according to LCS records; LCS-G 1–3: LCS-G 1–3: locomotion score groups 1 to 3; CPS: claw-position scoring.

	CPS in Total Numbers			
LCS-G	1	2	3	*n*
1	451	132	5	588
2	190	202	17	409
3	54	116	19	189

**Table 7:** Detailed results for parameters derived from AMS data according to LCS groups; LCS-G 1–3: locomotion score groups 1 to 3.

	Effects	*p*	LS Means	SE	*p*-Values for LCS-G ^1^	
Daily milkings (*n*)	LCS-G	<0.0001				
	1		3.24	±0.03	1 vs. 2	0.0291
	2		3.14	±0.03	2 vs. 3	0.0018
	3		2.93	±0.06	1 vs. 3	<0.0001
	Farm	<0.0001				
	Breed	0.1668				
	Lactation number	<0.0001				
	Lactation stage	<0.0001				
	First calving age	0.1880				
Daily milk yield (kg)	LCS-G	0.1913				
	1		26.7	±0.27	1 vs. 2	-
	2		26.5	±0.31	2 vs. 3	-
	3		25.7	±0.57	1 vs. 3	-
	Farm	<0.0001				
	Breed	<0.0001				
	Lactation number	<0.0001				
	Lactation stage	<0.0001				
	First calving age	0.0253				
Milking interval (minutes)	LCS-G	<0.0001				
	1		587	±4.85	1 vs. 2	0.0128
	2		603	±5.39	2 vs. 3	0.0001
	3		643	±9.99	1 vs. 3	<0.0001
	Farm	<0.0001				
	Breed	0.1215				
	Lactation number	<0.0001				
	Lactation stage	<0.0001				
	First calving age	0.0112				
Average milk production per hour (kg)	LCS-G	0.3300				
	1		1.23	±0.01	1 vs. 2	-
	2		1.23	±0.01	2 vs. 3	-
	3		1.20	±0.02	1 vs. 3	-
	Farm	<0.0001				
	Breed	<0.0001				
	Lactation number	<0.0001				
	Lactation stage	<0.0001				
	First calving age	0.0290				

^1^ Based on Tukey–Kramer test.

**Table 8:** Detailed results for sensor parameters according to LCS groups; LCS-G 1–3: locomotion score groups 1 to 3.

	Effects	*p*	LS Means	SE	*p*-Values for LCS-G ^1^	
Rumination (min/day)	LCS-G	0.95674				
	1		560	±2.38	1 vs. 2	-
	2		561	±2.66	2 vs. 3	-
	3		561	±4.92	1 vs. 3	-
	Farm	<0.0001				
	Breed	<0.0001				
	Lactation number	<0.0001				
	Lactation stage	0.0097				
	First calving age	<0.0001				
Eating (min/day)	LCS-G	<0.0001				
	1		281	±2.66	1 vs. 2	0.0001
	2		268	±2.96	2 vs. 3	0.0001
	3		245	±5.49	1 vs. 3	<0.0001
	Farm	<0.0001				
	Breed	<0.0001				
	Lactation number	<0.0001				
	Lactation stage	0.1560				
	First calving age	<0.0001				
Rest (min/day)	LCS-G	<0.0001				
	1		387	±3.51	1 vs. 2	<0.0001
	2		410	±3.91	2 vs. 3	<0.0001
	3		442	±7.25	1 vs. 3	<0.0001
	Farm	<0.0001				
	Breed	<0.0001				
	Lactation number	<0.0001				
	Lactation stage	<0.0001				
	First calving age	0.0473				
Activity_Mid (min/day)	LCS-G	<0.0001				
	1		148	±2.34	1 vs. 2	0.0062
	2		140	±2.61	2 vs. 3	0.0426
	3		128	±4.83	1 vs. 3	0.0001
	Farm	<0.0001				
	Breed	0.0252				
	Lactation number	<0.0001				
	Lactation stage	0.0081				
	First calving age	0.0016				
Activity_Trend (min/day)	LCS-G	<0.0001				
	1		305	±1.74	1 vs. 2	<0.0001
	2		291	±1.94	2 vs. 3	0.0002
	3		277	±3.59	1 vs. 3	<0.0001
	Farm	<0.0001				
	Breed	<0.0001				
	Lactation number	<0.0001				
	Lactation stage	<0.0001				
	First calving age	0.0314				

^1^ Based on Tukey–Kramer test.

**Table 9:** Mean ± standard deviation of model performance parameters from fourfold cross-validation. Model 1 was trained on sensor data only; Model 2 included AMS data, animal, and farm information; for Model 3 AMS, animal and farm data were merged with sensor records; and Model 4 additionally included BCS data. In Model 5, the CPS was included.

	Model 1	Model 2	Model 3	Model 4	Model 5
Sensitivity	0.610 (±0.009)	0.657 (±0.022)	0.695 (±0.030)	0.738 (±0.014)	0.725 (±0.090)
Specificity	0.640 (±0.005)	0.605 (±0.020)	0.668 (±0.041)	0.701 (±0.031)	0.775 (±0.025)
Accuracy	0.623 (±0.006)	0.629 (±0.020)	0.680 (±0.014)	0.719 (±0.010)	0.753 (±0.046)

**Table 10:** Mean ± standard deviation of model performance parameters for the respective number of cross-validations among the overall farm-based split under consideration of a low (<30%), medium (30–50%), and high (>50%) lameness incidence risk including animal and farm data, AMS data, and sensor records.

	<30%	30–50%	>50%
Sensitivity	0.743 (±0.021)	0.598 (±0.055)	0.454 (±0.034)
Specificity	0.486 (±0.088)	0.600 (±0.033)	0.685 (±0.117)
Accuracy	0.651 (±0.061)	0.599 (±0.022)	0.553 (±0.042)

**Table 11:** Mean ± standard deviation of model performance parameters for the respective number of cross-validations among each sub-dataset with a low (<30%), medium (30–50%), and high (>50%) lameness incidence risk including animal and farm data, AMS data, and sensor records.

	<30%	30–50%	>50%
Sensitivity	0.721 (±0.023)	0.585 (±0.048)	0.502 (±0.108)
Specificity	0.564 (±0.092)	0.603 (±0.047)	0.645 (±0.097)
Accuracy	0.687 (±0.020)	0.591 (±0.015)	0.574 (±0.064)

## Data Availability

The data used in the current study are not publicly available due to privacy restrictions of the data provider and owner (LKV Austria Gemeinnützige GmbH; https://lkv.at/ (accessed on 1 June 2022)). Authors were provided with anonymized data according to an authorized material transfer agreement. Supplementary data may be available upon reasonable request from the corresponding author.

## Supplementary Materials

The following supporting information can be downloaded at: https://www.mdpi.com/article/10.3390/ani13071180/s1, Figure S1: Mean hourly activity values (in minutes) for sensor parameter ‘Rumination’ showing no visible reduction for LCS-G 2 and LCS-G 3 cows; Figure S2: Mean hourly activity values (in minutes) for sensor parameter ‘Eating’ showing a visible reduction for LCS-G 2 and LCS-G 3 cows; Table S1: Questionnaire on farm management and husbandry conditions.

## References

[1] Bell M.J., Tzimiropoulos G. (2018). Novel Monitoring Systems to Obtain Dairy Cattle Phenotypes Associated With Sustainable Production. Front. Sustain. Food Syst..

[2] Borghart G.M., O’Grady L.E., Somers J.R. (2021). Prediction of lameness using automatically recorded activity, behavior and production data in post-parturient Irish dairy cows. Ir. Vet. J..

[3] Barkema H.W., Von Keyserlingk M.A., Kastelic J.P., Lam T.J.G.M., Luby C., Roy J.-P., Leblanc S.J., Keefe G.P., Kelton D.F. (2015). Invited review: Changes in the dairy industry affecting dairy cattle health and welfare. J. Dairy Sci..

[4] Garcia E., Klaas I., Amigo J.M., Bro R., Enevoldsen C. (2014). Lameness detection challenges in automated milking systems addressed with partial least squares discriminant analysis. J. Dairy Sci..

[5] King M.T.M., Pajor E.A., LeBlanc S.J., DeVries T.J. (2016). Associations of herd-level housing, management, and lameness prevalence with productivity and cow behavior in herds with automated milking systems. J. Dairy Sci..

[6] Westin R., Vaughan A., de Passillé A.M., DeVries T., Pajor E.A., Pellerin D., Siegford J., Vasseur E., Rushen J. (2016). Lying times of lactating cows on dairy farms with automatic milking systems and the relation to lameness, leg lesions, and body condition score. J. Dairy Sci..

[7] Tse C., Barkema H.W., DeVries T.J., Rushen J., Pajor E.A. (2017). Effect of transitioning to automatic milking systems on producers’ perceptions of farm management and cow health in the Canadian dairy industry. J. Dairy Sci..

[8] Galon N. (2010). The use of pedometry for estrus detection in dairy cows in Israel. J. Reprod. Dev..

[9] Bewley J.M., Schutz M.M. Recent studies using a reticular bolus system for monitoring dairy cattle core body temperature. Proceedings of the First North American Conference on Precision Dairy Management.

[10] Banhazi T.M., Lehr H., Black J.L., Crabtree H., Schofield P., Tscharke M., Berckmans D. (2012). Precision livestock farming: An international review of scientific and commercial aspects. Int. J. Agric. Biol. Eng..

[11] Bikker J.P., van Laar H., Rump P., Doorenbos J., van Meurs K., Griffioen G.M., Dijkstra J. (2014). Technical note: Evaluation of an ear-attached movement sensor to record cow feeding behavior and activity. J. Dairy Sci..

[12] Rutten C.J., Kamphuis C., Hogeveen H., Huijps K., Nielen M., Steeneveld W. (2017). Sensor data on cow activity, rumination, and ear temperature improve prediction of the start of calving in dairy cows. Comput. Electron. Agric..

[13] Gusterer E., Kanz P., Krieger S., Schweinzer V., Süss D., Lidauer L., Kickinger F., Öhlschuster M., Auer W., Drillich M. (2020). Sensor technology to support herd health monitoring: Using rumination duration and activity measures as unspecific variables for the early detection of dairy cows with health deviations. Theriogenology.

[14] Van Hertem T., Maltz E., Antler A., Romanini C.E.B., Viazzi S., Bahr C., Schlageter-Tello A., Lokhorst C., Berckmans D., Halachmi I. (2013). Lameness detection based on multivariate continuous sensing of milk yield, rumination, and neck activity. J. Dairy Sci..

[15] Van Nuffel A., Zwertvaegher I., Van Weyenberg S., Pastell M., Thorup V.M., Bahr C., Sonck B., Saeys W. (2015). Lameness detection in dairy cows: Part 2. Use of sensors to automatically register changes in locomotion or behavior. Animals.

[16] Beer G., Alsaaod M., Starke A., Schuepbach-Regula G., Müller H., Kohler P., Steiner A. (2016). Use of extended characteristics of locomotion and feeding behavior for automated identification of lame dairy cows. PLoS ONE.

[17] Alsaaod M., Fadul M., Steiner A. (2019). Automatic lameness detection in cattle. Vet. J..

[18] ZuchtData Jahresbericht (2021). Annual Report. https://www.zar.at/Downloads/Jahresberichte/ZuchtData-Jahresberichte.html/.

[19] Van De Gucht T., Van Weyenberg S., Van Nuffel A., Lauwers L., Vangeyte J., Saeys W. (2017). Supporting the development and adoption of automatic lameness detection systems in dairy cattle: Effect of system cost and performance on potential market shares. Animals.

[20] Kaniyamattam K., Hertl J., Lhermie G., Tasch U., Dyer R., Gröhn Y.T. (2020). Cost benefit analysis of automatic lameness detection systems in dairy herds: A dynamic programming approach. Prev. Vet. Med..

[21] Rouha-Mülleder C., Iben C., Wagner E., Laaha G., Troxler J., Waiblinger S. (2009). Relative importance of factors influencing the prevalence of lameness in Austrian cubicle loose-housed dairy cows. Prev. Vet. Med..

[22] Kofler J., Fürst-Waltl B., Dourakas M., Steininger F., Egger-Danner C. (2021). Impact of lameness on milk yield in dairy cows in Austria—Results from the Efficient-Cow-project. Schweiz. Arch. Tierheilkd..

[23] Kofler J., Suntinger M., Mayerhofer M., Linke K., Maurer L., Hund A., Fiedler A., Duda J., Egger-Danner C. (2022). Benchmarking based on regularly recorded claw health data of Austrian dairy cattle for implementation in the Cattle-Data-Network (RDV). Animals.

[24] Murray R.D., Downham D.Y., Clarkson M.J., Faull W.B., Hughes J.W., Manson F.J., Merritt J.B., Russell W.B., Sutherst J.E., Ward W.R. (1996). Epidemiology of lameness in dairy cattle: Description and analysis of foot lesions. Vet. Rec..

[25] Bruijnis M.R.N., Beerda B., Hogeveen H., Stassen E.N. (2012). Assessing the welfare impact of foot disorders in dairy cattle by a modeling approach. Animal.

[26] Whay H.R., Shearer J.K. (2017). The impact of lameness on welfare of the dairy cow. Vet. Clin. Food Anim. Pract..

[27] Bruijnis M.R.N., Hogeveen H., Stassen E.N. (2010). Assessing economic consequences of foot disorders in dairy cattle using a dynamic stochastic simulation model. J. Dairy Sci..

[28] Charfeddine N., Pérez-Cabal M.A. (2017). Effect of claw disorders on milk production, fertility, and longevity, and their economic impact in Spanish Holstein cows. J. Dairy Sci..

[29] Beggs D.S., Jongman E.C., Hemsworth P.H., Fisher A.D. (2019). Lame cows on Australian dairy farms: A comparison of farmer-identified lameness and formal lameness scoring, and the position of lame cows within the milking order. J. Dairy Sci..

[30] Sadiq M.B., Ramanoon S.Z., Shaik Mossadeq W.M., Mansor R., Syed Hussain S.S. (2019). Dairy farmers’ perceptions of and actions in relation to lameness management. Animals.

[31] Sprecher D.J., Hostetler D.E., Kaneene J.B. (1997). A lameness scoring system that uses posture and gait to predict dairy cattle reproductive performance. Theriogenology.

[32] Flower F.C., Weary D.M. (2006). Effect of hoof pathologies on subjective assessments of dairy cow gait. J. Dairy Sci..

[33] Van Nuffel A., Zwertvaegher I., Pluym L., Van Weyenberg S., Thorup V.M., Pastell M., Sonck B., Saeys W. (2015). Lameness detection in dairy cows: Part 1. How to distinguish between non-lame and lame cows based on differences in locomotion or behavior. Animals.

[34] Dunthorn J., Dyer R.M., Neerchal N.K., McHenry J.S., Rajkondawar P.G., Steingraber G., Tasch U. (2015). Predictive models of lameness in dairy cows achieve high sensitivity and specificity with force measurements in three dimensions. J. Dairy Res..

[35] Viazzi S., Bahr C., Van Hertem T., Schlageter-Tello A., Romanini C.E.B., Halachmi I., Lokhorst C., Berckmans D. (2013). Comparison of a three-dimensional and two-dimensional camera system for automated measurement of back posture in dairy cows. Comput. Electron. Agric..

[36] Jabbar K.A., Hansen M.F., Smith M.L., Smith L.N. (2017). Early and non-intrusive lameness detection in dairy cows using 3-dimensional video. Biosyst. Eng..

[37] Pluk A., Bahr C., Leroy T., Poursaberi A., Song X., Vranken E., Maertens W., Van Nuffel A., Berckmans D. (2010). Evaluation of step overlap as an automatic measure in dairy cow locomotion. Trans. ASABE.

[38] Van Nuffel A., Saeys W., Sonck B., Vangeyte J., Mertens K.C., De Ketelaere B., Van Weyenberg S. (2015). Variables of gait inconsistency outperform basic gait variables in detecting mildly lame cows. Livest. Sci..

[39] Chapinal N., de Passillé A.M., Weary D.M., von Keyserlingk M.A.G., Rushen J. (2009). Using gait score, walking speed, and lying behavior to detect hoof lesions in dairy cows. J. Dairy Sci..

[40] Lin Y.C., Mullan S., Main D.C.J. (2018). Optimising lameness detection in dairy cattle by using handheld infrared thermometers. Vet. Med. Sci..

[41] Van De Gucht T., Saeys W., Van Meensel J., Van Nuffel A., Vangeyte J., Lauwers L. (2018). Farm-specific economic value of automatic lameness detection systems in dairy cattle: From concepts to operational simulations. J. Dairy Sci..

[42] Poursaberi A., Bahr C., Pluk A., Van Nuffel A., Berckmans D. (2010). Real-time automatic lameness detection based on back posture extraction in dairy cattle: Shape analysis of cow with image processing techniques. Comput. Electron. Agric..

[43] Barker Z.E., Vázquez Diosdado J.A., Codling E.A., Bell N.J., Hodges H.R., Croft D.P., Amory J.R. (2018). Use of novel sensors combining local positioning and acceleration to measure feeding behavior differences associated with lameness in dairy cattle. J. Dairy Sci..

[44] Nechanitzky K., Starke A., Vidondo B., Müller H., Reckardt M., Friedli K., Steiner A. (2016). Analysis of behavioral changes in dairy cows associated with claw horn lesions. J. Dairy Sci..

[45] Bach A., Dinarés M., Devant M., Carré X. (2006). Associations between lameness and production, feeding and milking attendance of Holstein cows milked with an automatic milking system. J. Dairy Res..

[46] Miguel-Pacheco G.G., Kaler J., Remnant J., Cheyne L., Abbott C., French A.P., Pridmore T.P., Huxley J.N. (2014). Behavioural changes in dairy cows with lameness in an automatic milking system. Appl. Anim. Behav. Sci..

[47] Egger-Danner C., Fuerst-Waltl B., Obritzhauser W., Fuerst C., Schwarzenbacher H., Grassauer B., Mayerhofer M., Koeck A. (2012). Recording of direct health traits in Austria-Experience report with emphasis on aspects of availability for breeding purposes. J. Dairy Sci..

[48] Edmonson A.J., Lean I.J., Weaver L.D., Farver T., Webster G. (1989). A body condition scoring chart for Holstein dairy cows. J. Dairy Sci..

[49] Bulgarelli-Jiménez G., Dercks K., Van Amerongen J., Schukken Y.H., Nielsen M. A hind feet position scoring system to monitor subclinical lameness in Dutch Holstein-Friesian cows. Proceedings of the 9th International Symposium on Disorders of the Ruminant Digit and the International Conference on Lameness in Cattle.

[50] Holzhauer M., Middelesch H., Bartels C.J.M., Frankena K., Verhoeff J., Noordhuizen-Stassen E.N., Noordhuizen J.P.T.M. (2005). Assessing the repeatability and reproducibility of the Leg Score: A Dutch claw health scoring system for dairy cattle. Tijdschr. Diergeneeskd..

[51] Fuerst-Waltl B., Egger-Danner C., Guggenbichler S., Kofler J. (2021). Impact of lameness on fertility traits in Austrian Fleckvieh cows—Results from the Efficient-Cow-project. Schweiz. Arch. Tierheilkd..

[52] Landis J.R., Koch G.G. (1977). An application of hierarchical kappa-type statistics in the assessment of majority agreement among multiple observers. Biometrics.

[53] R Core Team (2021). R: A Language and Environment for Statistical Computing.

[54] Lenth R.V. (2016). Least-Squares Means: The R Package lsmeans. J. Stat. Softw..

[55] Blackie N., Amory J., Bleach E., Scaife J. (2011). The effect of lameness on lying behaviour of zero grazed Holstein dairy cattle. Appl. Anim. Behav. Sci..

[56] Breiman L. (2001). Random forests. Mach. Learn..

[57] Cutler A., Cutler D.R., Stevens J.R. (2012). Random Forests. Ensemble Machine Learning.

[58] Ho T.K. Random decision forests. Proceedings of the 3rd International Conference on Document Analysis and Recognition.

[59] Han H., Guo X., Yu H. Variable selection using Mean Decrease Accuracy and Mean Decrease Gini based on Random Forest. Proceedings of the 7th IEEE International Conference on Software Engineering and Service Science (ICSESS).

[60] Wright M.N., Ziegler A. (2017). Ranger: A fast implementation of random forests for high dimensional data in C++ and R. J. Stat. Softw..

[61] Kuhn M., Wing J., Weston S., Williams A., Keefer C., Engelhardt A., Cooper T., Mayer Z., Kenkel B., The R Core Team (2021). Caret: Classification and Regression Training. https://CRAN.R-project.org/package=caret.

[62] Coelho Ribeiro L.A., Bresolin T., Rosa G.J.D.M., Rume Casagrande D., Danes M.D.A.C., Dórea J.R.R. (2021). Disentangling data dependency using cross-validation strategies to evaluate prediction quality of cattle grazing activities using machine learning algorithms and wearable sensor data. J. Anim. Sci..

[63] Wang Q., Bovenhuis H. (2019). Validation strategy can result in an overoptimistic view of the ability of milk infrared spectra to predict methane emission of dairy cattle. J. Dairy Sci..

[64] Yunta C., Guasch I., Bach A. (2012). Short communication: Lying behavior of lactating dairy cows is influenced by lameness especially around feeding time. J. Dairy Sci..

[65] King M.T.M., LeBlanc S.J., Pajor E.A., DeVries T.J. (2017). Cow-level associations of lameness, behavior, and milk yield of cows milked in automated systems. J. Dairy Sci..

[66] Foditsch C., Oikonomou G., Machado V.S., Bicalho M.L., Ganda E.K., Lima S.F., Rossi R., Ribeiro B.L., Kussler A., Bicalho R.C. (2016). Lameness prevalence and risk factors in large dairy farms in upstate New York—Model development for the prediction of claw horn disruption lesions. PLoS ONE.

[67] Von Keyserlingk M.A.G., Barrientos A., Ito K., Galo E., Weary D.M. (2012). Benchmarking cow comfort on North American freestall dairies: Lameness, leg injuries, lying time, facility design, and management for high-producing Holstein dairy cows. J. Dairy Sci..

[68] Bell M.J., Wall E., Russell G., Roberts D.J., Simm G. (2010). Risk factors for culling in Holstein-Friesian dairy cows. Vet. Rec..

[69] Weigele H.C., Gygax L., Steiner A., Wechsler B., Burla J.B. (2018). Moderate lameness leads to marked behavioral changes in dairy cows. J. Dairy Sci..

[70] Thorup V.M., Nielsen B.L., Robert P.-E., Giger-Reverdin S., Konka J., Michie C., Friggens N.C. (2016). Lameness affects cow feeding but not rumination behavior as characterized from sensor data. Front. Vet. Sci..

[71] Norring M., Häggman J., Simojoki H., Tamminen P., Winckler C., Pastell M. (2014). Short communication: Lameness impairs feeding behavior of dairy cows. J. Dairy Sci..

[72] Pothmann H., Erlen A., Pichler M., Drillich M. (2015). Relationship and repeatability of body condition scoring and backfat thickness measurement in dairy cows by different investigators. Berlin. Munch. Tierarztl. Wochenschr..

[73] King M.T.M., Sparkman K.J., LeBlanc S.J., DeVries T.J. (2018). Milk yield relative to supplement intake and rumination time differs by health status for fresh cows milked with automated systems. J. Dairy Sci..

[74] Lasser J., Matzhold C., Egger-Danner C., Fuerst-Waltl B., Steininger F., Wittek T., Klimek P. (2021). Integrating diverse data sources to predict disease risk in dairy cattle—A machine learning approach. J. Anim. Sci..

[75] Chen T., Guestrin C. Xgboost: A scalable tree boosting system. Proceedings of the 22nd acm sigkdd international conference on knowledge discovery and data mining. 13–17 August 2016.

[76] Werema C.W., Yang D.A., Laven L.J., Mueller K.R., Laven R.A. (2022). Evaluating alternatives to locomotion scoring for detecting lameness in pasture-based dairy cattle in New Zealand: In-Parlour scoring. Animals.

[77] Herzberg D., Strobel P., Ramirez-Reveco A., Werner M., Bustamante H. (2020). Chronic inflammatory lameness increases cytokine concentration in the spinal cord of dairy cows. Front. Vet. Sci..

[78] Jewell M.T., Cameron M., Spears J., McKenna S.L., Cockram M.S., Sanchez J., Keefe G.P. (2019). Prevalence of lameness and associated risk factors on dairy farms in the Maritime Provinces of Canada. J. Dairy Sci..

[79] Browne N., Hudson C.D., Crossley R.E., Sugrue K., Kennedy E., Huxley J.N., Conneely M. (2022). Cow-and herd-level risk factors for lameness in partly housed pasture-based dairy cows. J. Dairy Sci..

[80] Browne N., Hudson C.D., Crossley R.E., Sugrue K., Kennedy E., Huxley J.N., Conneely M. (2022). Lameness prevalence and management practices on Irish pasture-based dairy farms. Ir. Vet. J..

[81] Matzhold C., Lasser J., Egger-Danner C., Fuerst-Waltl B., Wittek T., Kofler J., Steininger F., Klimek P. (2021). A systematic approach to analyse the impact of farm-profiles on bovine health. Sci. Rep..

