# Supplementary material for: The Combined Use of Automated Milking System and Sensor Data to Improve Detection of Mild Lameness in Dairy Cattle
Source: Animals (Basel). 2023 Mar 28;13(7):1180. doi: 10.3390/ani13071180 (PMC10093521; doi:10.3390/ani13071180)
Supplement: Supplementary file 1 [file animals-13-01180-s001.zip › animals-2289157-supplementary.pdf]

Table S1: Questionnaire on farm management and husbandry conditions.

N	Question	Answers	Included in model
**General Information**			
1	Farm run as full-time or spare-time job?	• Full-time<br>• Spare-time	No, all farmers working full-time
2	Conventional or organic farm?	• Conventional<br>• Organic	Yes
**Feeding**			
3	Time for rumen adjustment when changing silage?	• < 5 days<br>• > 5 days	Yes
4	Proportion of concentrate?	• > 45%<br>• 35 – 45%<br>• < 35%	Yes
5	Proportion of structured feed?	• No calculation<br>• ___ %	Yes
6	Is the length of structured feed particles above 3cm?	• Yes<br>• No	No, all farmers answered Yes
7	Are feed analyses done?	• Yes, mostly<br>• Yes, sometimes<br>• No	Yes
8	Are feed rations calculated throughout the year?	• Yes<br>• No	Yes
9	Addition of minerals and vitamins to the ration?	• No<br>• Yes, for cows in milk<br>• Yes, for dry cows<br>• Yes, for youngstock	Yes
10	How is the concentrate fed?	• Individually<br>• Per group<br>• Mixed form	No, as all farms feed an upgraded mixed ration at the feeding trough and individually assign the remaining concentrate over the AMS
11	Which animal groups share the same feed ration?	• Early-lactating cows<br>• Late-lactating cows<br>• Dry cows<br>• Youngstock < 1 year<br>• Youngstock > 1 year	No, only cows in milk were used in the final model and early-lactating and late-lactating animals receive the same feed ration on all farms.
12	Was the feeding management changed within the last few years?	• Yes<br>• No	No, no changes during study period
**Husbandry**			
13	Is there alpine pasture?	• No<br>• Yes, for cows in milk<br>• Yes, for dry cows<br>• Yes, for youngstock	Yes

14	Are cooling systems (ventilator, air tubes) available?	• No<br>• Yes, for cows in milk<br>• Yes, for dry cows<br>• Yes, for youngstock	Yes
15	Are cow showers available?	• No<br>• Yes, for cows in milk<br>• Yes, for dry cows<br>• Yes, for youngstock	Yes
16	Are animals kept on pasture?	• No<br>• Yes, for cows in milk<br>• Yes, for dry cows<br>• Yes, for youngstock	Yes
17	If animals are kept on pasture, what type of procedure?	• Whole day<br>• At night<br>• Two times a day<br>• other	Yes
18	For how many days are animals kept on pasture?	• ___	Yes
19	How many hectares of pasture are available?	• ___	Yes
20	Do cows have access to outdoor areas?	• Yes, paved outdoor area<br>• Yes, but not paved<br>• No	Yes
21	Do they have permanent access?	• Yes<br>• No	No, as all farms with outdoor areas give permanent access
22	How are animals kept in tie-stalls?	• No tie-stall<br>• Other methods	No, all cows kept in free stall barns
23	How are animals added to the herd?	• Abruptly<br>• Visual contact beforehand<br>• differing	Yes
24	Are there calving pens?	• Yes<br>• No	No, all farms have calving pens
25	How many cows are kept in the calving pen?	• One cow<br>• More cows	Yes
26	How often is the calving pen cleaned?	• After each calving<br>• Less often	No, no calving pen is cleaned after each calving
27	For how long are cows kept in the calving pen after giving birth?	• ___ days	Yes
28	For how many days before calving are heifers added to the herd?	• ___ days<br>• Only after calving<br>• All animals are kept in the same group	Yes
**Hoof trimming**			
29	On which animals is hoof trimming performed?	• All cows in milk and dry cows	Yes

		• All cows in milk without dry cows<br>• Only lame cows<br>• All heifers<br>• Only lame heifers<br>• others	
30	Are footbaths used?	• Yes<br>• No	No, no footbaths used
31	How often are animals scored for lameness?	• 1-2 times per month<br>• 1-2 times per month with documentation<br>• never	No, all farmers answered the same
**Barn Measurements**			
32	Inclined feeding fence?	• Yes<br>• No	Yes
33	Cubicle Bedding Height?	• Measured on four cubicles on three different places	Yes
34	Cubicle Bed Length?	• Measured on four cubicles	Yes
35	Cubicle Width?	• Measured on four cubicles	Yes
36	Curb?	• Measured on four cubicles	Yes
37	Brisket Board?	• Measured on four cubicles	Yes
38	Height of Neck rail?	• Measured on four cubicles	Yes
39	Neck rail Diagonal?	• Measured on four cubicles	Yes
40	Abnormal lying behavior in cows?	• Yes<br>• No	Yes
41	Damaged flooring?	• Yes<br>• No	Yes
42	Slip-resistant flooring?	• Yes<br>• Partly<br>• No	Yes
43	Type of flooring?	• Slatted<br>• Solid<br>• Rubber mats	Yes
44	Steps or obstacles within the barn?	• Yes<br>• No	Yes
45	Width of feeding alleys?	• ___	Yes
46	Width of walking alleys?	• ___	Yes
47	Number of drinkers/troughs?	• ___	Yes
48	Are areas for lying down clean?	• Yes, more than 2/3 are clean	Yes

		• No, more than 1/3 is dirty	
49	Are animals clean?	• Yes, more than 2/3 are clean<br>• No, more than 1/3 is dirty	Yes
50	How many cows have injuries or swollen joints?	• ___ %	Yes

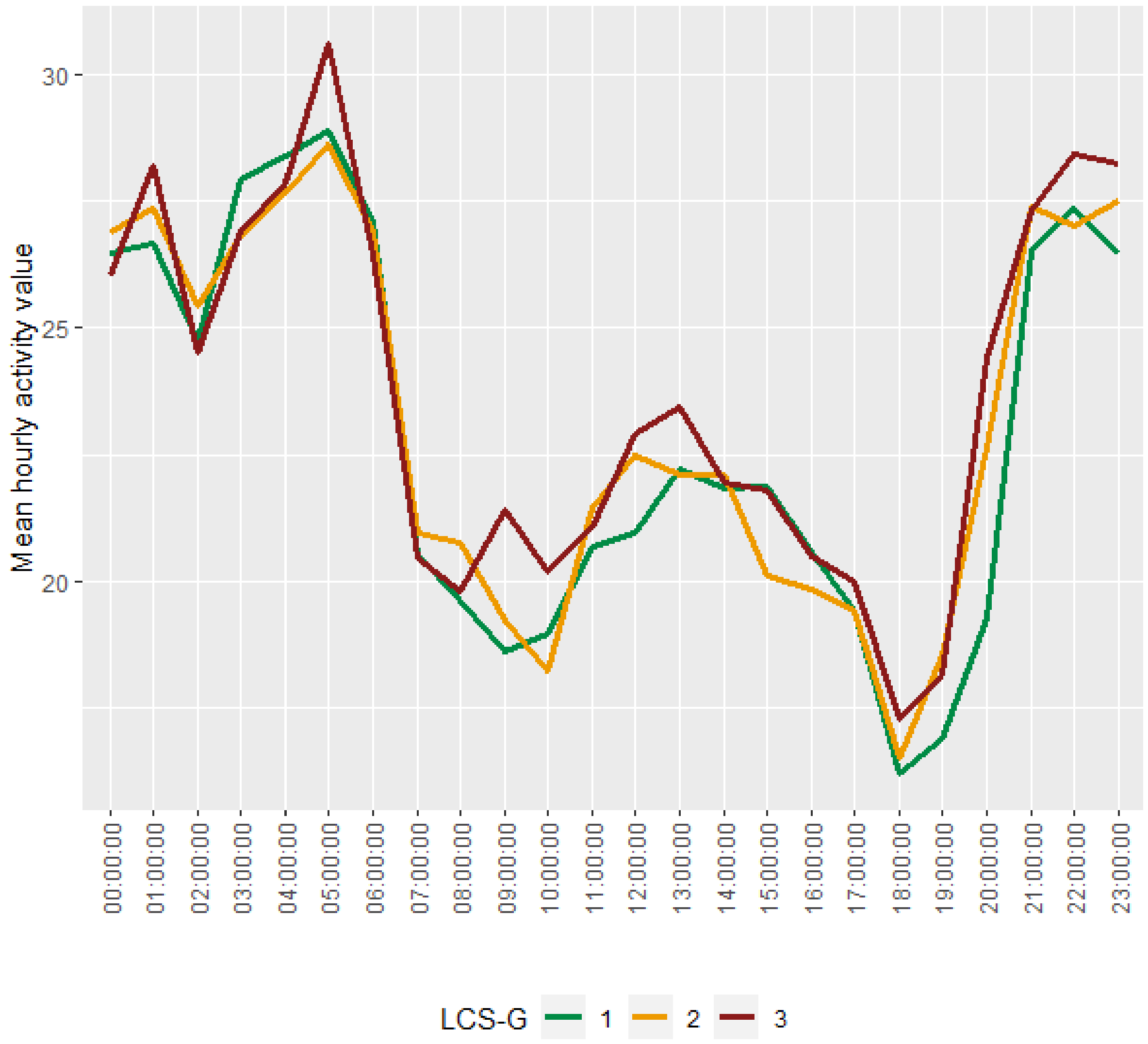

Figure S1: Mean hourly activity values (in minutes) for sensor parameter 'Rumination' showing no visible reduction for LCS-G 2 and LCS-G 3 cows.

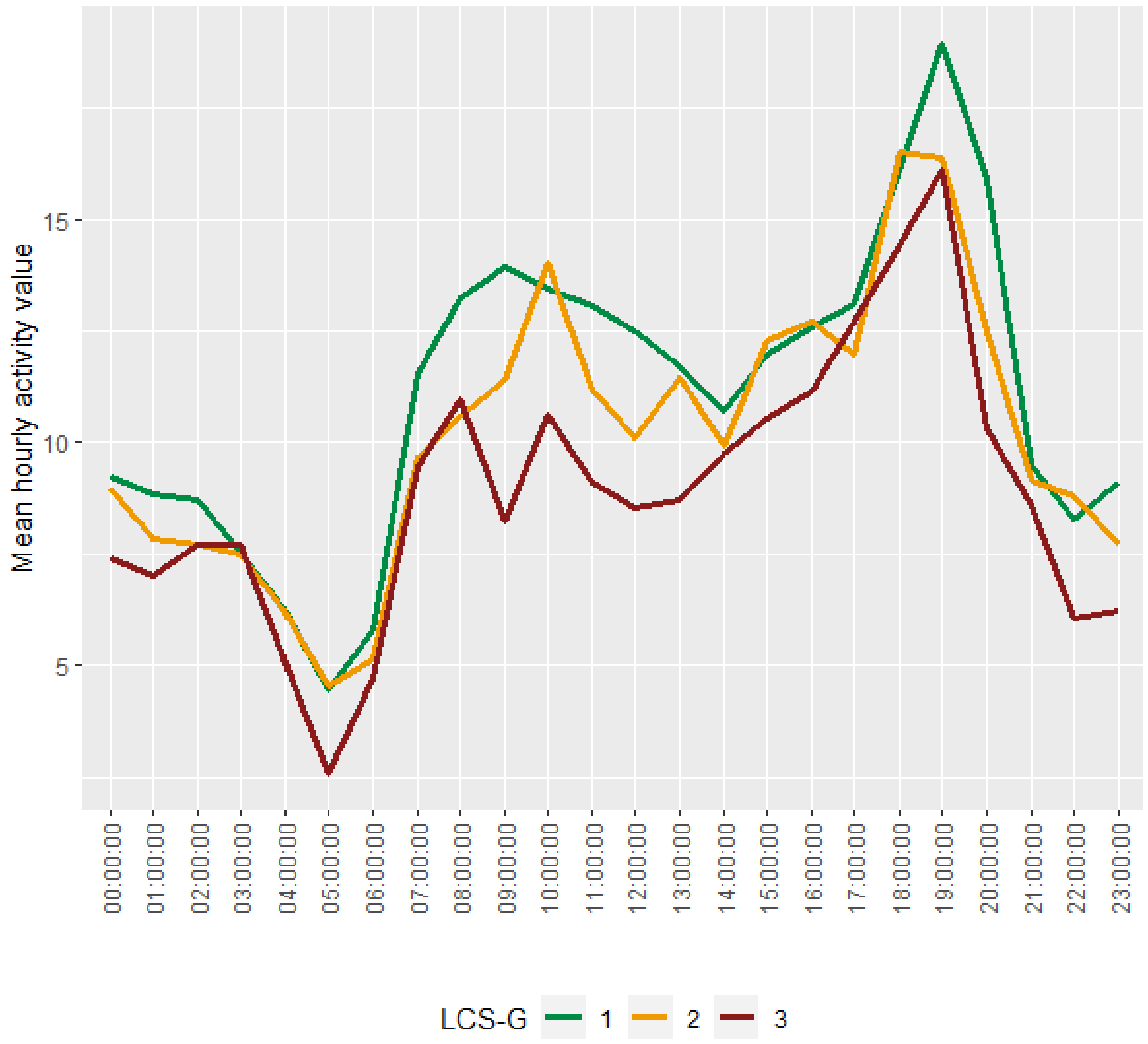

Figure S2: Mean hourly activity values (in minutes) for sensor parameter 'Eating' showing a visible reduction for LCS-G 2 and LCS-G 3 cows.
